# Feasibility, Acceptability, and Preliminary Outcomes of a Cognitive Behavioral Therapy–Based Mobile Mental Well-being Program (Noom Mood): Single-Arm Prospective Cohort Study

**DOI:** 10.2196/36794

**Published:** 2022-04-15

**Authors:** Meaghan McCallum, Annabell Suh Ho, Ellen Siobhan Mitchell, Christine N May, Heather Behr, Lorie Ritschel, Kirk Mochrie, Andreas Michaelides

**Affiliations:** 1 Academic Research Noom Inc New York, NY United States; 2 Department of Integrative Health Saybrook University Pasadena, CA United States; 3 Triangle Area Psychology Clinic Durham, NC United States; 4 School of Medicine University of North Carolina Chapel Hill, NC United States

**Keywords:** mHealth, mobile mental health, mental health, stress, anxiety

## Abstract

**Background:**

The prevalence of anxiety, depression, and general distress has risen in recent years. Mobile mental health programs have been found to provide support to nonclinical populations and may overcome some of the barriers associated with traditional in-person treatment; however, researchers have voiced concerns that many publicly available mobile mental health programs lack evidence-based theoretical foundations, peer-reviewed research, and sufficient engagement from the public.

**Objective:**

This study aimed to evaluate the feasibility, acceptability, and preliminary outcomes of Noom Mood, a commercial mobile cognitive behavioral therapy– and mindfulness-based program.

**Methods:**

In this single-arm prospective cohort study, individuals who joined Noom Mood between August and October 2021 completed surveys at baseline and 4-week follow-up. Per-protocol analyses included those who completed both surveys (n=113), and intention-to-treat analyses included all participants (N=185).

**Results:**

A majority of the sample reported that the program is easy to use, they felt confident recommending the program to a friend, and they perceived the program to be effective at improving stress and anxiety. There were significant improvements in anxiety symptoms, perceived stress, depressive feelings, emotion regulation, and optimism in both the per-protocol and intention-to-treat analyses (all *P*<.001). Participants reported benefiting most from learning skills (eg, breathing and cognitive reframing techniques), interacting with the program features, and gaining awareness of their emotions and thought patterns. Participants also made a number of suggestions to improve product functionality and usability.

**Conclusions:**

Results suggest that Noom Mood is feasible and acceptable to participants, with promising preliminary outcomes. Future studies should build on these results to evaluate the effects of Noom Mood using more rigorous designs.

## Introduction

The World Health Organization stresses the importance of mental health, which they broadly define as a state of “well-being in which an individual realizes his or her own abilities and can cope with the normal stresses of life” [1]. Many individuals are affected by difficulties with mental health [2]; for example, anxiety disorders are highly prevalent worldwide and are estimated to affect 18% of individuals in the United States alone [3,4]. Lifetime prevalence for depression is approximately 17% [2]. Furthermore, it is increasingly recognized that the general population can benefit from mental health support, regardless of whether clinical thresholds for mental illness are met [5,6]: as many as 57% to 84% of US adults have reported subclinical but substantial amounts of stress or worry in recent years [7,8]. Estimates suggest that anxiety, depression, and stress are associated with greater risk of mortality and hundreds of billions of dollars in economic burden per year [9,10]. 

Although a number of empirically supported treatments for mental health difficulties are available, myriad barriers exist that make it difficult for many people to access traditional in-person support, including cost, long waiting times to see providers, and limited provider availability, especially for individuals living in remote areas [11-15]. The COVID-19 pandemic has also increased barriers to accessing in-person support, potentially increasing willingness to seek digital support [16,17]. In addition, many individuals avoid seeking treatment due to stigma or to mistrust of the mental health system more generally [11,13].

In recent years, there has been a proliferation of interest in and development of mobile mental health programs. Use of these programs has tripled in recent years [18], and multiple reviews suggest that mobile mental health apps have the capacity to improve mental health and emotion regulation in the general population [19,20]. Mobile mental health has the potential to address many of the aforementioned barriers to treatment [21,22]; perhaps most importantly, mobile mental health allows for support or psychoeducation that is not restricted by time, location, or provider availability. In addition, digital (ie, via smartphone) delivery increases accessibility and autonomy in allowing for largely self-directed care [5,23]. Such programs facilitate self-monitoring of mood or activity, a well-known strategy to change undesired behaviors [5]. Lastly, mobile platforms allow for objective measurement of behavioral indicators, such as the number of articles read, and, therefore, allow individuals to track which strategies are most effective in helping them achieve behavioral change.

Despite this proliferation of readily accessible mobile mental health programs, researchers have raised several concerns that merit attention and that can be viewed through the lens of implementation science (see Proctor et al [24] for an in-depth discussion of implementation science variables as they apply to outcome studies). First, many mobile health (mHealth) programs available to the public are not based on evidence-based theoretical frameworks [25]. Moreover, users are self-selected, meaning that the problems they are experiencing may or may not map onto the content including the mobile app (ie, problems with appropriateness) [25]. Second, whether evidence based or not, many programs are used briefly and then discarded (ie, problems with adoption) or do not reach a broad enough segment of the population to be useful (ie, problems with penetration) [18,26]. Research has found that thousands of programs have been released on app stores that retain a very limited number of active users over time; for example, studies have shown that 97% of users do not use these mental health apps at day 15 [26,27]. This represents an obvious challenge for mental health programs, as intervention engagement has been associated with better outcomes in a multitude of studies [28-30]. Lastly, few of these mobile mental health programs include a research component to evaluate feasibility, acceptability, or outcomes of any sort; of programs based on theoretical frameworks, only approximately 6.2% have associated peer-reviewed research [25,31,32].

As such, this study was designed to address these gaps in the literature by examining the feasibility, acceptability, and preliminary outcomes of Noom Mood, a widely available commercial mHealth program that incorporates evidence-based recommendations for mobile mental health programs [5]. In particular, this study aims to contribute to the substantial gap in the evidence base, identified by implementation science researchers and review papers on mobile mental health, in data from commercial programs [25,31,33,34]. Another contribution of this study stems from Noom Mood’s inclusion of personal coaching for guidance and implementation of cognitive behavioral therapy (CBT) techniques, but not clinical therapy. Few studies have examined widely available mental health programs guided by personal coaching; many existing studies examine mental health programs that are entirely self-guided (ie, without individualized coaching support), are designed to provide clinical therapy or serve as an adjunct to therapy, or provide personalized coaching in other contexts (eg, employer-provided coaching or for specific conditions) [35-38]. 

**Figure 1:**
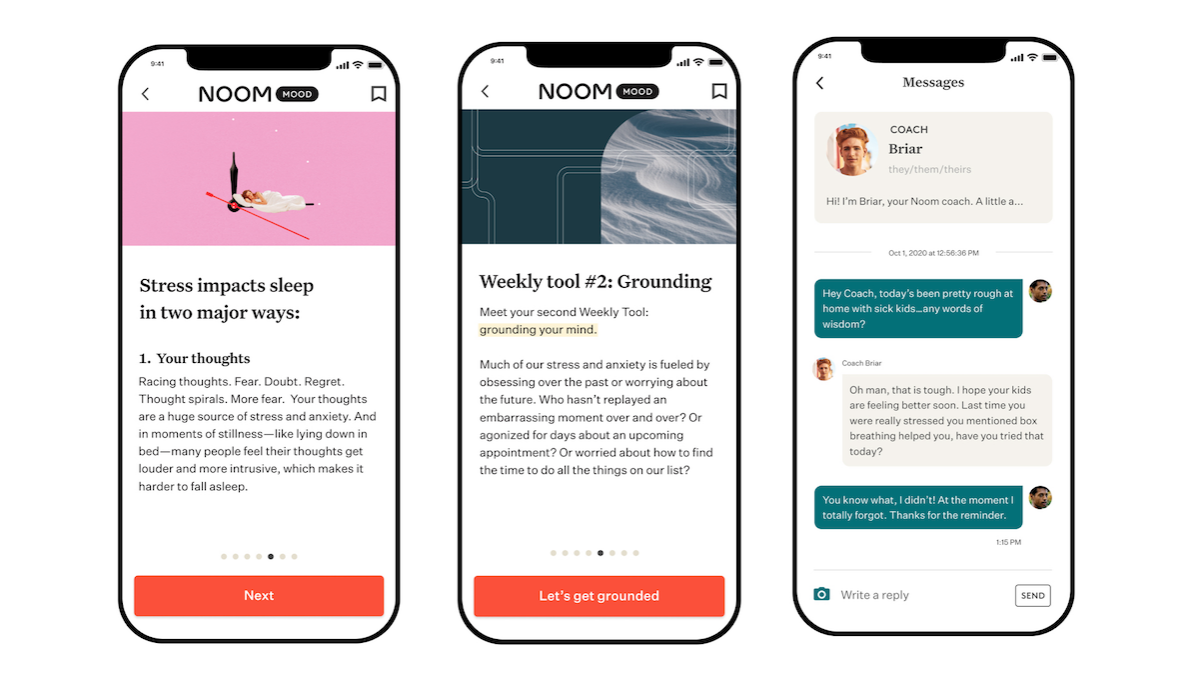
Screenshots of the Noom Mood program.

Noom Mood is a structured, skills-based approach to stress and anxiety management*.* Noom Mood uses strategies from empirically supported treatments that have been shown to improve mental health outcomes, such as anxiety, depression, and stress (eg, CBT, dialectical behavior therapy [DBT], acceptance and commitment therapy [ACT], and mindfulness-based stress reduction [MBSR]) [39-43]. Importantly, preliminary evidence has shown that CBT and MBSR can be deployed on a mobile platform and that these programs are associated with improvements in mental well-being in nonclinical and clinical populations [23,44]; however, as described previously, more empirical evaluation is needed of evidence-based, commercial programs. Program components include the following: (1) a daily curriculum consisting of psychoeducational articles for users to read, (2) individualized coaching offered through in-app messaging, (3) weekly skills-based activities, and (4) a mood-logging feature. All four components are expected to improve mental well-being (eg, reduce perceived anxiety and depressive symptoms and perceived stress). The curriculum, activities, and coaching were derived from evidence-based frameworks (ie, CBT, DBT, ACT, and MBSR) that have been shown to be effective in improving these outcomes, so these three components would be expected to be most directly related to outcomes. The fourth component of mood logging is based on behavior change techniques of self-monitoring, helping users to build self-awareness of their mood and associated behaviors [45]. More specifically, the daily curriculum was developed in collaboration with clinical psychologists and was designed to translate evidence-based treatments and psychoeducation into a format that is useful for individuals within a self-help framework. For example, each day, participants are presented with a short article that explains conceptual terms and principles (eg, cognitive defusion from ACT), provides practical tips and quizzes to build knowledge, and guides users through a relevant practical activity (eg, how to practice cognitive defusion over the next week; Figure 1). Because of the utility of skills-training activities that help to apply evidence-based principles into daily life [5,46,47], Noom Mood introduces individuals to a short 10- to 15-minute practical activity based on evidence-based frameworks, such as breathing techniques and cognitive reframing at the beginning of each week. The activity is implemented for 1 week, with a practice on day 7 in which individuals reflect on the skill learned and how well it worked for them (Figure 1). Lastly, Noom Mood includes a messaging feature that allows participants to communicate directly with health coaches (Figure 1). Coaches help users to understand and engage in activities, encourage reflection and awareness of patterns, and provide validation for emotional experiences based on CBT techniques. Coaching protocols were adapted to this mental well-being context from the Noom weight management program, for which coaching has been refined and tested and shown to provide guidance on activities, emotional self-awareness, and emotional validation [48]. Noom Mood coaches are trained in CBT techniques but are not licensed clinicians, as Noom Mood does not provide clinical assessment, diagnoses, or treatment and is not a replacement for therapy. The coaching feature was included to address concerns that have been cited in previous studies of evidence-based programs [48-50]. Specifically, human contact from remote coaches within otherwise self-guided digital programs may encourage engagement and improve outcomes [48,51,52]. One randomized controlled trial (RCT) found that engagement check-ins from coaches improved engagement in a web-based depression program [53].

The first step in evaluating any new mHealth platform is to investigate stakeholders’ views on the feasibility and acceptability of the proposed product [24,50]. Feasibility is defined as the extent to which end users feel that they could and would use the product in their lives for the purposes for which it was designed [54]. Acceptability is defined as the extent to which stakeholders find the product satisfactory with regard to its content and perceived credibility [54]. Results from feasibility and acceptability testing are then used to refine and update the platform to align with stakeholders’ suggestions more closely. 

The primary goal of this study was to evaluate the feasibility and acceptability of Noom Mood, as well as to gather preliminary data on whether the program might be associated with improved well-being. We hypothesized that users would find the platform to be feasible and acceptable. Furthermore, we hypothesized that participants who used the program would report some benefit in terms of improved anxiety symptoms, stress, depressive feelings, emotion regulation, and optimism by the end of the 4-week study.

## Methods

A single-arm prospective cohort design was used to test feasibility and acceptability of Noom Mood, as well as initial symptom and well-being outcomes.

### Ethics Approval

The study was approved by the Advarra Institutional Review Board (protocol No. 00055306).

### Procedure and Participants

Participants were recruited from the pool of individuals who had voluntarily signed up for the Noom Mood program. A randomly selected subset of adults who voluntarily enrolled in the Noom Mood program between August and October 2021 were invited to participate. All participants provided informed consent prior to participation. Inclusion criteria for participants were as follows: located within the United States, English speaking, and aged 18 years or older. Participants were invited to complete the baseline questionnaire within 1 business day of signing up for the Noom Mood program. Those who completed the baseline questionnaire were invited to complete the follow-up survey 4 weeks later. Study completers were compensated with a US $20 gift card for their participation. Participants did not receive the program for free during or after the study. The entire study occurred remotely, including online administration of surveys via email.

### Noom Mood Program

The Noom Mood program was deployed as described above. At the time of this study, approximately 15 psychoeducational articles were presented to participants each week. In addition to the curriculum, participants had access to mood-logging features, and they were encouraged by coaches to engage in the curriculum and to log their mood once per day.

### Measures

#### Feasibility

Feasibility was assessed at 4-week follow-up.

##### System Usability Scale

The System Usability Scale (SUS) [55] is a 10-item scale assessing stakeholders’ views of ease of use. Items were modified to substitute “program” for “system.” Participants were asked to rate their agreement with each usability statement (eg, “I thought the program was easy to use”) on a scale of 1 (“strongly disagree”) to 5 (“strongly agree”). After reverse-scoring relevant items, sum scores were multiplied by 2.5 to create a final score ranging from 0 to 100. Research indicates that SUS scores above 68 are considered above average and scores below 68 are below average. Internal reliability for the SUS was excellent (α=.90).

##### Program Engagement Data

As in past work [56], feasibility was also evaluated via the amount of time participants spent engaging with the program. Engagement data consisted of usage and self-report data recorded by the program for 4 weeks. Self-report and usage data were collected by the mobile program and stored on a secured cloud server from Amazon Web Services [57]. Data were deidentified prior to extraction from the database. Engagement measures included the frequency with which participants completed mood logs, number of times the app was opened, number of articles read, number of messages sent to the coach, and number of activities completed. Data were also extracted to evaluate the number of days the user was active, which was defined as the number of days with at least one in-app action. In order to measure real-world engagement, participants were not given specific minimum engagement requirements to remain in the study.

#### Acceptability

Acceptability was assessed at 4-week follow-up. 

##### Credibility and Expectancy Questionnaire

The Credibility and Expectancy Questionnaire (CEQ) [58] is a 6-item scale that was originally designed to assess perceptions of treatment credibility and expectancy for improvement in psychotherapy. To render the scale more appropriate for use in this study, questionnaire items were modified slightly (ie, “program” was substituted for “therapy” and “stress and anxiety” was substituted for “symptoms”). Items in the CEQ range either from 1 to 9 or from 0 to 100, depending on the item. In line with the CEQ’s factor structure and following previous work [59], we computed average credibility and expectancy scores reflected by the first three and last three items of the scale, respectively. Internal reliability was excellent (credibility subscale: α=.90; expectancy subscale: α=.93).

##### Program Satisfaction Questionnaire

We asked the following open-ended questions: (1) What is the main benefit you received from Noom’s stress and anxiety management program? (2) How can we improve Noom’s stress and anxiety management program for you? (3) What was the most helpful part of the program? and (4) What was the least helpful part of the program? Because of the variety of answers possible, content analysis was used to code each response into categories and calculate the percentage of responses allocated to each category. The categories were created using latent Dirichlet allocation (LDA), a machine learning approach for automatic clustering of text data [60]. LDA is an unsupervised approach that automatically identifies latent clusters of words (ie, categories) that cluster within unclassified data. Each word cluster was assigned a label, or category name, by a master coder with experience with the program. For each question, each participant response was given a score (0 or 1) for each category since one response could apply to multiple categories. Interrater reliability between the master coder and another coder blind to the study’s hypotheses and design ranged from 0.72 to 1.0 for all categories, suggesting good to excellent reliability [61].

#### Symptom and Well-being Outcomes

Symptom and well-being outcomes were assessed at baseline and 4-week follow-up.

##### 7-Item Generalized Anxiety Disorder Scale

The 7-item Generalized Anxiety Disorder scale (GAD-7) [62] is a 7-item scale that assesses the extent to which individuals experience symptoms of anxiety (eg, “Feeling nervous, anxious, or on edge”) on a scale of 0 (“not at all”) to 3 (“nearly every day”). Internal reliability for the GAD-7 was good (α=.82 and α=.87 for baseline and follow-up, respectively).

##### 4-Item Perceived Stress Scale

The 4-item Perceived Stress Scale (PSS-4) [63] is a 4-item scale assessing the frequency with which individuals experience various symptoms of stress (eg, “How often have you felt that you were unable to control the important things in your life?”) on a scale of 0 (“never”) to 4 (“very often”). Internal reliability for the PSS-4 was adequate (α=.68 and α=.69 for baseline and follow-up, respectively).

##### 8-Item Patient Health Questionnaire Depression Scale

The 8-item Patient Health Questionnaire depression scale (PHQ-8) [64] is an 8-item scale that assesses the extent to which participants experience feelings of depression (eg, “feeling down, depressed, or hopeless” or “little interest or pleasure in doing things”) on a scale of 0 (“not at all”) to 3 (“nearly every day”). Internal reliability for the PHQ-8 was good (α=.84 and α=.85 for baseline and follow-up, respectively).

##### Difficulties in Emotion Regulation Scale–Short Form

The Difficulties in Emotion Regulation Scale–Short Form (DERS-SF) [65,66] is an 18-item scale assessing emotion dysregulation. It comprises six subscales: emotional awareness, clarity about the nature of one’s emotions, acceptance of one’s emotions, access to effective emotion regulation strategies, ability to engage in goal-directed activities while experiencing negative emotions, and ability to manage one’s impulses during negative emotions. These subscales (α=.74-.91 and α=.76-.91) and the DERS-SF total score (α=.89 at both time points) demonstrated good internal consistency at baseline and follow-up, respectively.

##### Life Orientation Test–Revised

The Life Orientation Test–Revised (LOT-R) [67] is a 10-item scale that assesses trait optimism. Individuals are asked to rate their agreement with each statement (eg, “In uncertain times, I usually expect the best.”) on a scale of 0 (“strongly disagree”) to 4 (“strongly agree”). Internal reliability for the LOT-R was good (α=.86 and α=.85 at baseline and follow-up, respectively).

### Statistical Analysis

Analyses were conducted in SPSS software (version 27; IBM Corp). For acceptability and feasibility, survey responses were descriptively analyzed with mean scores and percentages of participants that chose each response. For open-ended acceptability responses, content-analyzed categories are presented descriptively with the percentage of responses that fall into each category. Descriptive statistics were also conducted for engagement measures to evaluate feasibility. For preliminary outcomes, paired 2-tailed *t* tests were conducted to evaluate changes on all quantitative variables from baseline to week 4. Both per-protocol and intention-to-treat analyses were conducted. The per-protocol sample consisted of participants who completed both assessments (n=113) and included those who started the program but stopped using it. Intention-to-treat analyses included data from all participants who began the study (N=185); baseline scores were carried forward for participants who did not complete the week-4 assessment. Effect sizes were calculated using Cohen *d* [68].

## Results

### Participant Characteristics

Participants’ demographic characteristics are presented in Table 1. A total of 185 unique Noom Mood users enrolled in the study and completed the baseline survey. Of these, 113 (62.1%) participants completed the follow-up survey. Participants who completed both baseline and follow-up surveys did not differ significantly from those who completed only the baseline survey in terms of any demographic variables or baseline survey values.

**Table 1:** Participant characteristics.

Demographics			Per-protocol sample (n=113)		Intention-to-treat sample (N=185)
Age (years), mean (SD)			36.8 (9.8)		37.3 (10.4)
**Gender, n (%)**					
	Male	15 (13.3)		32 (17.3)	
	Female	94 (83.2)		141 (76.2)	
	Other	2 (1.8)		3 (1.6)	
	Prefer not to say or N/A^a^	2 (1.8)		9 (4.9)	
**Ethnicity, n (%)**					
	Hispanic	13 (11.5)		20 (10.8)	
	Not Hispanic	97 (85.8)		153 (82.7)	
	Prefer not to say or N/A	3 (2.7)		12 (6.5)	
**Race, n (%)**					
	White	99 (87.6)		153 (82.7)	
	Black or African American	5 (4.4)		7 (3.8)	
	Asian or Pacific Islander	3 (2.7)		11 (5.9)	
	Other	0 (0)		1 (0.5)	
	Prefer not to say or N/A	6 (5.3)		13 (7.0)	
**Employment status, n (%)**					
	Employed	88 (77.8)		144 (77.8)	
	Not employed	12 (10.6)		18 (9.7)	
	Retired	1 (0.9)		2 (1.1)	
	Disabled	5 (4.4)		6 (3.2)	
	Student	5 (4.4)		6 (3.2)	
	Prefer not to say or N/A	2 (1.8)		9 (4.9)	
**Education, n (%)**					
	High school, GED^b^, or less education	7 (6.2)		10 (5.4)	
	Some college or associate degree	24 (21.2)		37 (20.0)	
	College graduate	45 (39.8)		67 (36.2)	
	Graduate degree	35 (31.0)		62 (33.5)	
	Prefer not to say or N/A	2 (1.8)		9 (4.9)	

^a^N/A: not applicable.

^b^GED: General Education Development.

### Feasibility

Responses to the SUS are presented in Table 2. As noted above, scores of 68 or higher on the SUS indicate above-average ratings of system usability. A majority (79/109, 72.5%) of participants had overall system usability scores of 68 or higher (mean 77.40, SD 19.45), which is considered an indication of good usability [59]. Most participants reported that the program was easy to use (85/110, 77.3%), and they thought that other people would be able to learn to use the program very quickly (93/109, 85.3%).

Program engagement data are presented in Table 3. Engagement data are presented as weekly averages (ie, the number of times the participant engaged in the behavior over the course of the study divided by the total number of weeks). Participants engaged within the app several times per week on average. Over 4 weeks, the per-protocol sample averaged 14.1 (SD 9.02) app opens, with 2 mean app opens per week. They had an average of 12.1 days with an in-app action, amounting to 1.7 active days per week. The intention-to-treat sample opened the app, on average, 13.7 (SD 8.6) times over 4 weeks, with an average of 1.96 app opens per week. They completed at least one in-app action on an average of 11.2 (SD 8.7) days, which amounted to 1.6 active days per week.

**Table 2:** Participants reporting good feasibility and acceptability.

Survey measure^a^			Value
**System Usability Scale item, n (%)**			
	I would like to use this program frequently. (n=109)	62 (56.9)	
	I found the program unnecessarily complex.^b^ (n=110)	81 (73.6)	
	I thought the program was easy to use. (n=110)	85 (77.3)	
	I would need the support of a technical person to be able to use this program.^b^ (n=108)	95 (88.0)	
	I found the various functions in this program were well integrated. (n=109)	76 (69.7)	
	I thought there was too much inconsistency in the program.^b^ (n=109)	87 (79.8)	
	I would imagine that most people would learn to use this program very quickly. (n=109)	93 (85.3)	
	I found the program very cumbersome to use.^b^ (n=109)	77 (70.6)	
	I felt very confident using the program. (n=108)	78 (72.2)	
	I needed to learn a lot of things before I could get going with the program.^b^ (n=107)	89 (83.2)	
System Usability Scale score of 68 or higher (n=109), n (%)			79 (72.5)
System Usability Scale overall score, mean (SD)			77.4 (19.4)
**Credibility and Expectancy Questionnaire item, n (%)**			
	At this point, how logical does the program seem to you? (n=110)	101 (91.8)	
	How successful do you think this program was in reducing stress and anxiety? (n=109)	83 (76.1)	
	How confident would you be in recommending this program to a friend who experiences stress and anxiety? (n=108)	87 (80.6)	
	By the end of the program, how much improvement in stress and anxiety do you think will occur?^c^ (n=108)	63 (58.3)	
	At this point, how much do you really feel that the program will help to reduce stress and anxiety? (n=108)	85 (78.7)	
	By the end of this program, how much improvement in stress and anxiety do you feel will occur?^c^ (n=107)	62 (57.9)	

^a^The table includes participants who chose 4 or greater (out of 5) on the System Usability Scale or 5 or greater (out of 9) on the Credibility and Expectancy Questionnaire, except where indicated.

^b^These participants chose 2 or less (out of 5) on the System Usability Scale.

^c^These participants chose at least 50% out of 100%.

**Table 3:** Average total engagement over 4 weeks.

Type of engagement	Per-protocol sample (n=110)^a^, mean (SD)	Intention-to-treat sample (n=181)^a^, mean (SD)
App opens	14.11 (9.02)	13.72 (8.60)
Articles read	37.12 (28.87)	34.39 (27.44)
Mood logs	10.59 (9.54)	10.00 (10.79)
Days with one in-app action	12.14 (9.03)	11.24 (8.68)
Messages sent to coach	9.71 (10.33)	8.24 (9.09)
Activities^b^	1.33 (2.68)	1.30 (2.54)

^a^Sample sizes represent all participants for whom matching data from the database could be identified.

^b^Activities were calculated over 3 weeks because one offline activity was not tracked by the program.

### Acceptability

Responses to the CEQ are presented in Table 2. Of note, the table displays the frequency and percentage of participants who chose at least a 5 (“somewhat”) out of 9 (“very much”) on the CEQ. The vast majority of participants (101/110, 91.8%) rated the program as at least somewhat logical (mean 7.1, SD 1.9, range 1-9). Most (83/109, 76.1%) thought the program was at least somewhat successful at reducing stress and anxiety (mean 5.6, SD 2.2, range 1-9). Many participants (87/108, 80.6%) also felt at least somewhat confident in recommending the program to a friend (mean 6.1, SD 2.3, range 1-9). Most participants (85/108, 78.7%) felt the program would help to reduce stress and anxiety at least somewhat (mean 5.7, SD 2.3), with more than half (63/108, 58.3%) expecting it to reduce their stress or anxiety by 50% or more (mean 4.9, SD 2.5, with 0 referring to 0% and 10 referring to 100%).

Responses to the Program Satisfaction Questionnaire are presented in Table 4. Participants reported benefiting most from the skills and techniques they learned or practiced (eg, breathing techniques and thought reframing; 38/106, 35.8%). Participants also reported benefiting from the program’s features or capabilities (eg, mood tracking and articles; 31/106, 29.2%) and greater awareness (eg, learning and reflection) encouraged by the program (30/106, 28.3%). Specifically, participants found the articles (18/106, 17.0%), coaching (18/106, 17.0%), and qualities of the program (eg, manageable, convenient, and “great” attitude; 16/106, 15.1%) to be the most helpful parts of Noom Mood.

For potential areas of improvement, most participants did not provide a response or indicated that they had no suggested improvements (37/106, 34.9%). The next most common response was “other” (21/106, 19.8%), or participants requested a new feature or program idea (19/106, 17.9%). “Other” responses included increasing the frequency of reminders, expanding areas of content (eg, support for procrastination), and slowing the pace of tasks. Participants also preferred a lower cost (16/106, 15.1%), with some mentioning the potential to be reimbursed, as well as a more personalized experience (9/106, 8.5%) and greater flexibility (9/106, 8.5%), such as the ability to progress while skipping articles, accessing future articles, or repeating an activity for another week.

When asked to describe the least helpful parts of Noom Mood, most participants did not provide a response (40/106, 37.7%). The next most common response was “other” (21/106, 19.8%); responses noted that the program contained too much repetition and that the pacing of the program needed improvement. Lastly, some participants (17/106, 16.0%) described coaching as the least helpful aspect of the program, noting that they would prefer to interact with a coach with specialized expertise or to receive more personalized responses.

**Table 4:** Response frequencies in each category for the Program Satisfaction Questionnaire.

Category		Participant responses (n=106), n (%)^a^
**Main benefit of Noom Mood**		
	Skills and techniques	38 (35.8)
	Program features or capabilities	31 (29.2)
	Awareness (ie, learning and reflection)	30 (28.3)
	Emotional experience and management	27 (25.5)
	Other	18 (17.0)
	None or no response	15 (14.2)
**Areas to improve**		
	None or no response	37 (34.9)
	Other	21 (19.8)
	New feature or program idea	19 (17.9)
	Cost	16 (15.1)
	Coaching	15 (14.2)
	Personalization	9 (8.5)
	Flexibility	9 (8.5)
	Articles	6 (5.7)
	Activities	3 (2.8)
**Most helpful part of Noom Mood**		
	Coaching	18 (17.0)
	Articles	18 (17.0)
	None or no response	17 (16.0)
	Qualities of the program	16 (15.1)
	Skills and techniques	15 (14.2)
	Activities	14 (13.2)
	Awareness (ie, learning and reflection)	9 (8.5)
	Other	9 (8.5)
	Mood tracking	5 (4.7)
	Everything	3 (2.8)
**Least helpful part of Noom Mood**		
	None or no response	40 (37.7)
	Other	21 (19.8)
	Coaching	17 (16.0)
	Activities	9 (8.5)
	Mood tracking	7 (6.6)
	Articles	6 (5.7)
	Personalization and interactivity	5 (4.7)
	Cost	4 (3.8)
	Everything	2 (1.9)

^a^Each response could be placed in more than one category. Categories were derived from individuals’ open-ended responses.

### Symptom and Well-being Outcomes

From baseline to 4 weeks, there was a significant reduction in anxiety symptoms for both per-protocol samples (Table 5; t_112_=10.92, *P*<.001, *d*=1.03) and intention-to-treat samples (t_184_=9.48, *P*<.001, *d*=0.70) with large and medium effect sizes, respectively. There was also a significant improvement in perceived stress (per-protocol sample: t_112_=7.69, *P*<.001, *d*=0.72; intention-to-treat sample: t_184_=7.09, *P*<.001, *d*=0.52) and depressive feelings (per-protocol sample: t_110_=7.88, *P*<.001, *d*=0.75; intention-to-treat sample: t_181_=7.40, *P*<.001, *d*=0.55) with medium effect sizes. Finally, there were significant improvements in emotion regulation (per-protocol sample: t_105_=5.93, *P*<.001, *d*=0.58; intention-to-treat sample: t_178_=5.79, *P*<.001, *d*=0.43) and optimism (per-protocol sample: t_104_=–5.04, *P*<.001, *d*=–0.49; intention-to-treat sample: t_175_=–5.15, *P*<.001, *d*=–0.39) with small to medium effect sizes. 

**Table 5:** Symptom and well-being outcomes from baseline to 4 weeks.

Outcome	Per-protocol sample (n=113)^a^						Intention-to-treat sample (N=185)^b^				
	Baseline, mean (SD)	4 weeks, mean (SD)	ΔMean (% change)^c^	*P* value	Effect size^d^	Baseline, mean (SD)		4 weeks, mean (SD)	ΔMean (% change)^c^	*P* value	Effect size^d^
Anxiety symptoms (GAD-7^e^)	13.30 (4.31)	8.54 (4.61)	–4.76 (–35.81)	<.001	1.03	13.28 (4.39)		10.18 (5.14)	–3.10 (–23.32)	<.001	0.70
Perceived stress (PSS-4^f^)	8.96 (2.39)	7.08 (2.29)	–1.88 (–21.03)	<.001	0.72	8.89 (2.41)		7.73 (2.48)	–1.16 (–13.07)	<.001	0.52
Depressive feelings (PHQ-8^g^)	11.67 (5.47)	7.77 (4.98)	–3.90 (–33.39)	<.001	0.75	11.99 (5.55)		9.40 (5.66)	–2.59 (–21.61)	<.001	0.55
Emotion regulation (DERS-SF^h^)	45.97 (11.86)	39.39 (11.30)	–6.57 (–14.30)	<.001	0.58	47.01 (13.09)		42.95 (13.49)	–4.05 (–8.63)	<.001	0.43
Optimism (LOT-R^i^)	7.05 (3.49)	8.16 (3.15)	1.11 (15.75)	<.001	0.49	7.33 (3.57)		8.09 (3.39)	0.75 (10.30)	<.001	0.39

^a^Per-protocol analyses only included participants who completed both survey assessments.

^b^For intention-to-treat analyses, baseline responses were carried forward for nonresponders.

^c^Negative values indicate decreases compared to baseline.

^d^Effect sizes constitute Cohen *d*.

^e^GAD-7: 7-item Generalized Anxiety Disorder scale.

^f^PSS-4: 4-item Perceived Stress Scale.

^g^PHQ-8: 8-item Patient Health Questionnaire depression scale.

^h^DERS-SF: Difficulties in Emotion Regulation Scale–Short Form; negative values on the DERS-SF indicate better emotional regulation (ie, fewer difficulties with emotional regulation).

^i^LOT-R: Life Orientation Test–Revised; positive values on the LOT-R indicate more optimism.

## Discussion

### Principal Findings

In reviews of mental health programs, researchers have voiced concerns about limited published research on commercial programs, and that programs either have limited public engagement or are not based on evidence-based theory [18,25-27,31,32]. Given the identified need for evidence from this type of commercial program [25,31], this pilot study evaluated the feasibility, acceptability, and preliminary outcomes of Noom Mood, which is widely publicly available, based on CBT and MBSR techniques, designed to encourage engagement among the general public, and includes personal coaching. Our results suggest that the program was usable, feasible, and acceptable to participants. In addition, self-reported anxiety symptoms, stress, depressive feelings, emotion regulation, and optimism improved from baseline to 4 weeks.

### Feasibility and Acceptability

#### Feasibility

Overall, participants rated the program as feasible. The average system usability score was 77.4, which surpasses the threshold for good usability [69], and more than 75% of participants reported that the program was easy to use. These scores are in line with feasibility and usability scores from other mobile programs [70-73]. Similar to levels of engagement reported in studies of comparable mobile mental health programs [46,70,74], participants in this study engaged with Noom Mood regularly, opening the program approximately two times per week and performing an action within the app once every 2 to 3 days (11 of 28 days). Participants engaged most with the articles and least with activities. Of note, it is possible that participants completed activities offline throughout the week, which is how they were designed, but did not mark them as complete in the app. As such, it is likely that the data collected on activities underestimate participant engagement in this aspect of Noom Mood, given that many activities focus on offline experiences (eg, practicing breathing exercises or grounding techniques). Future studies will aim to assess actions completed offline in relationship to symptom outcomes.

#### Acceptability

The vast majority of participants found the program to be logical (92%) and effective at reducing stress and anxiety (76%). Importantly, 81% of participants felt confident in recommending the program to a friend. These findings are similar to other studies of mobile mental health programs and suggest that the program was perceived to be acceptable to users [35,70,73]. Additionally, at the follow-up assessment, more than half of the participants reported that they expected that the program would eventually reduce their stress or anxiety by an additional 50% or more. Future work should investigate long-term outcomes and whether these participant expectations are borne out.

Participants reported benefiting most from skills training; program features such as articles, activities, and coaching; learning to better manage their emotions; and reflective processes such as learning, reflecting, and increasing their awareness. Participants reported benefiting from taking the time to reflect on how they were feeling and increasingly becoming aware of their emotions and thought patterns. Many participants also mentioned benefiting from the structure and accountability of a designated program. Participants appreciated the overall tenor of the program; one participant reflected that “the attitude it strikes is a great balance of cheeky humor but realistic so it’s not overly strict nor overly cheesy. Makes me connect with it well and stick with it.” Other participants, however, reported that they hoped for a more serious tone to the articles. At the time of the study, the program incorporated jokes and hashtags for the sake of relatability, and has since been modified in response to participant feedback. 

Participants also indicated that the program could be improved to better help individuals progress in a way that best suits an individual’s idiosyncratic wants or needs. For example, some participants wanted a slower pace, whereas others requested more daily reminders. Additionally, some participants provided feedback that they wanted more specialized interactions with coaches. While individuals were informed that Noom Mood is not a replacement for therapy and does not provide clinical assessment or treatment, it is possible that participants were expecting the coaching feature to function more similarly to therapy. However, some participants provided feedback stating that responses given by coaches did not feel personalized and felt too generic. It is also possible that some participants may not have been good candidates for a self-help approach. As mentioned previously, in the literature, there is limited understanding of how participants would experience a commercial mobile mental health program with personal coaching, rather than therapy. This study contributes initial understanding that, in this context, coaching can be helpful, but it can also raise confusion about the role of a coach when providing guidance and support rather than therapy. Future iterations of the program should, thus, be sure to set expectations for this feature clearly. 

Participants also relayed some suggestions for program improvements that would provide support in varying environments or situations, such as support for moms with young children, skills to reduce procrastination, video and audio recordings, and easily accessible summaries of activities or articles, all of which should be considered in future programs. Since the time of the study, audio recordings have been added to the program. Some participants reported that they would prefer that the program be offered at a lower cost, and some mentioned they would like the program to be covered by health insurance plans. In order to increase accessibility, future initiatives and programs should consider efforts to provide reimbursable experiences (eg, through employee wellness initiatives).

### Preliminary Outcomes

#### Anxiety Symptoms, Perceived Stress, and Depressive Feelings

From baseline to 4 weeks, anxiety symptoms improved by 36% (*d*=1.03) in per-protocol analyses and 23% (*d*=0.70) in intention-to-treat analyses. In addition, stress reductions were 21% (*d*=0.72, per-protocol analysis) and 13% (*d*=0.52, intention-to-treat analysis), and depressive feelings decreased by 33% (*d*=0.75, per-protocol analysis) and 22% (*d*=0.55, intention-to-treat analysis). These effect sizes are comparable to those reported in studies of other mobile mental health programs with the same study length and outcome measures [44,75-80]. Specifically, anxiety and stress decreased in ways that were comparable to or greater than anxiety reductions shown in previous studies, whereas depression showed comparable, though smaller, effect sizes [44,75,78,79]. Of course, this may reflect the fact that the program focuses more on stress and anxiety management than on depression. Of all our outcome measures, anxiety showed the biggest effect sizes, which contrasts with some studies that have found that anxiety scores did not improve as much as other symptom measures, such as depression [75,80].

#### Emotion Regulation and Optimism 

In this study, we found that emotion regulation improved by 14% (*d*=0.58, per-protocol analysis) and 8.6% (*d*=0.43, intention-to-treat analysis). Emotion dysregulation is hypothesized to underpin a wide range of psychological difficulties [81]; in fact, transdiagnostic interventions, such as DBT or the Unified Protocol [82], focus on emotion dysregulation as the primary treatment target. Notably, however, emotion regulation is rarely included as an outcome variable in mobile mental health programs, despite its empirical and theoretical relevance to mental health and well-being [19]. In two studies of mHealth programs conducted with young adults [83] and homeless youth [84] that measured emotion regulation as an outcome variable, results showed no significant improvements in emotion regulation capacity. 

We found significantly higher optimism at 4 weeks compared to baseline (15.7% or *d*=0.49, per-protocol analysis; 10% or *d*=0.39, intention-to-treat analysis). To our knowledge, this is the first mobile mental health study to measure changes in optimism, though some studies of mobile mental health programs have found improvements in other positive psychological constructs, such as life satisfaction, general mental well-being, or quality of life [46,85-88]. A robust literature base demonstrates that optimism is inversely correlated with depression and anxiety and positively correlated with measures of life satisfaction and self-reported health variables [89,90]. Importantly, optimism may influence physical and mental health by encouraging adaptive coping [85]. Consistent with previous findings, both baseline and 4-week optimism scores were significantly negatively correlated with time-matched anxiety symptoms, stress, and depressive feelings, and optimism scores were positively correlated with emotion regulation (ie, higher optimism is correlated with greater capacity to regulate one’s emotions). Future studies should evaluate optimism and its associations with other mental health outcomes.

### Limitations

This pilot study had several limitations. First, without a control group, it was not possible to separate the effects of the program itself from improvement over time (ie, regression to the mean and maturation). In addition, other interventions were uncontrolled; that is, program participants may have been participating in active therapy or may have been taking psychotropic medications while they were participating in this study. Nevertheless, it is unlikely that these findings are purely spurious, as the effect sizes are similar to those found in active treatment groups in RCTs, and they are much larger than those found in control groups (eg, see Bakker et al [75]). Now that preliminary feasibility and acceptability have been established, future studies should use randomized designs to confirm that these results were due to the program itself. Also, the study was conducted over 4 weeks, and it is unclear whether results would change over longer periods of time. Further, the study examined the program as a whole, making it difficult to isolate which specific program components led to changes in outcomes. Future studies should use causal methods to explore this further. In addition, the sample was primarily female, White, and highly educated, which is typical of studies of mobile mental health programs [19]. Future research should evaluate to what extent these results would generalize to other populations and actively recruit from hard-to-reach populations. Lastly, this study did not assess other variables that may have caused improvement in symptoms, such as psychiatric services, individual or group therapy, and participants’ use of other self-help materials.

### Conclusions

In this study, we explored the usability, feasibility, acceptability, and preliminary effectiveness of Noom Mood, a publicly available, mobile mental well-being program based on CBT and MBSR with personal coaching. The program follows 11 of Bakker et al’s [5] evidence-based recommendations for mobile mental health programs: it is based on CBT; addresses both anxiety and low mood; is designed for use by nonclinical populations; includes reporting of thoughts, feelings, and behaviors; recommends activities; provides mental health information; encourages non–technology-based activities; includes gamification or intrinsic motivation to engage; shows logs of past app use (eg, patterns of logged mood); uses reminders to engage (eg, messages from the coach); and provides a simple and intuitive interface and interactions. Our results suggest that Noom Mood was usable, feasible, and acceptable to participants, with promising preliminary improvements in anxiety symptoms, stress, depressive feelings, emotion regulation, and optimism. Future directions should include (1) the incorporation of changes suggested by participants in this study and (2) more rigorous testing of outcome variables, such as through randomized designs.

## Abbreviations

ACT: acceptance and commitment therapy
CBT: cognitive behavioral therapy
CEQ: Credibility and Expectancy Questionnaire
DBT: dialectical behavior therapy
DERS-SF: Difficulties in Emotion Regulation Scale–Short Form
GAD-7: 7-item Generalized Anxiety Disorder scale
LDA: latent Dirichlet allocation
LOT-R: Life Orientation Test–Revised
MBSR: mindfulness-based stress reduction
mHealth: mobile health
PHQ-8: 8-item Patient Health Questionnaire depression scale
PSS-4: 4-item Perceived Stress Scale
RCT: randomized controlled trial
SUS: System Usability Scale
